# Development of *Rhodococcus opacus* as a chassis for lignin valorization and bioproduction of high-value compounds

**DOI:** 10.1186/s13068-019-1535-3

**Published:** 2019-08-05

**Authors:** Winston E. Anthony, Rhiannon R. Carr, Drew M. DeLorenzo, Tayte P. Campbell, Zeyu Shang, Marcus Foston, Tae Seok Moon, Gautam Dantas

**Affiliations:** 1 The Edison Family Center for Genome Sciences and Systems Biology, Washington University in St. Louis School of Medicine, St. Louis, MO 63110 USA; 2 Department of Energy, Environmental and Chemical Engineering, Washington University in St. Louis, St. Louis, MO 63130 USA; 3 Department of Pathology and Immunology, Washington University in St. Louis School of Medicine, St. Louis, MO 63108 USA; 4 Department of Biomedical Engineering, Washington University in St. Louis, St. Louis, MO 63130 USA; 5 Department of Molecular Microbiology, Washington University in St. Louis School of Medicine, St. Louis, MO 63108 USA

**Keywords:** *Rhodococcus opacus* PD630, Non-model organism, Lignocellulose, Lignin, Biofuel, Bioproduct, Aromatic compound, Genetic tool, Thermochemical conversion, Biological conversion

## Abstract

The current extraction and use of fossil fuels has been linked to extensive negative health and environmental outcomes. Lignocellulosic biomass-derived biofuels and bioproducts are being actively considered as renewable alternatives to the fuels, chemicals, and materials produced from fossil fuels. A major challenge limiting large-scale, economic deployment of second-generation biorefineries is the insufficient product yield, diversity, and value that current conversion technologies can extract from lignocellulose, in particular from the underutilized lignin fraction. *Rhodococcus opacus* PD630 is an oleaginous gram-positive bacterium with innate catabolic pathways and tolerance mechanisms for the inhibitory aromatic compounds found in depolymerized lignin, as well as native or engineered pathways for hexose and pentose sugars found in the carbohydrate fractions of biomass. As a result, *R. opacus* holds potential as a biological chassis for the conversion of lignocellulosic biomass into biodiesel precursors and other value-added products. This review begins by examining the important role that lignin utilization will play in the future of biorefineries and by providing a concise survey of the current lignin conversion technologies. The genetic machinery and capabilities of *R. opacus* that allow the bacterium to tolerate and metabolize aromatic compounds and depolymerized lignin are also discussed, along with a synopsis of the genetic toolbox and synthetic biology methods now available for engineering this organism. Finally, we summarize the different feedstocks that *R. opacus* has been demonstrated to consume, and the high-value products that it has been shown to produce. Engineered *R. opacus* will enable lignin valorization over the coming years, leading to cost-effective conversion of lignocellulose into fuels, chemicals, and materials.

## Introduction

In comparison with estimated pre-industrial levels (circa 1700 CE), the current global atmospheric CO_2_ concentration has increased over 100 parts per million (ppm) and is now stably maintained over 400 ppm, with three quarters of that change occurring since 1960 due to emissions from the burning of fossil [1]. This increase in the CO_2_ concentration has already contributed to a small, but significant, rise in global average temperatures, and will lead to even greater increases in the future. Climate change can lead to decreasing crop yields and seed quality [1], facilitate sea level rise, promote destructive extreme weather events, and cause spikes in energy usage as increasingly frequent severe weather can cause unscheduled shutdown/start-up cycles [2]. Addressing greenhouse gas-driven climate change will require a complex, multi-tiered approach toward a more carbon–neutral world, including a greater usage of biofuels in the transportation industry and more sustainable chemical and material synthesis.

Biofuels or bioproducts, derived from biological sources (i.e., biomass) rather than petroleum, are not a new concept—the inventor of the diesel engine advocated for farmers generating their own vegetable-oil fuel in areas lacking a consistent source of petroleum [3]. First-generation biofuels and bioproducts are derived from food crops like corn, soy, palm, and sugarcane [3–6]. While these biofuels and bioproducts have the potential to mitigate CO_2_ emissions associated with fossil fuels [3, 5, 6], they are economically and environmentally problematic: energy uses related to irrigation, fertilizer production, cultivation, and transportation are significant, and global demand for food outweighs the supply of arable land [4, 6]. Other sources of biomass, like lignocellulose, are readily available as by-products of the agriculture and forestry industries. Moreover, the dedicated cultivation of properly selected and/or engineered species as sources of lignocellulose can be achieved in a wider distribution of climate and soil conditions with reduced water and fertilizer requirements compared to first-generation sources [7]. However, lignocellulose does require more complex processing to produce a second-generation biofuel or bioproduct. The structural component of lignocellulose, lignin, provides a particular challenge as it is a complex aromatic macromolecule that evolved to resist degradation [8]. Complete utilization and upgrading of lignin are critical for economic viability of second-generation biorefineries.

The processing of lignocellulosic biomass can be split up into two steps: (1) depolymerization in which the polymers within lignocellulose (e.g., cellulose, hemicellulose, and lignin) undergo cleavage reaction producing their respective subunits, and (2) upgrading in which these subunits are converted into a value-added product. Both steps of biomass processing can be achieved through diverse biological, thermochemical, and catalytic processes. However, the generation of a single value-added product requires upgrading followed by extensive chemical separations, or the use of biological catalysts (i.e., a microbe) that can funnel and convert many different lignocellulose-derived substrates into a single product. *Rhodococcus opacus* PD630 (hereafter *R. opacus*) has been identified as a potential biological chassis for the funneling and conversion of lignocellulose-derived substrates to lipids, a biofuel precursor. *R. opacus* has a natural tolerance to toxic aromatic compounds found in the lignin fraction of lignocellulose, an ability to increase this tolerance through adaption, and numerous catabolic pathways for consumption of both carbohydrates and aromatics, making it an ideal candidate to address the challenges of biomass conversion [9]. *R. opacus* has also shown the ability to accumulate up to ~ 80% of its cell dry weight in lipids, such as triacylglycerols (TAGs), under certain growth conditions. These lipids can then be converted to biodiesel via a transesterification reaction [10, 11]. In this review, we focus on discussing the important role of lignin valorization in regard to the viability of second-generation biorefineries, summarize different lignocellulose depolymerization methods, and examine *R. opacus*’ potential for the conversion of biomass breakdown products into diverse fuels and chemicals.

## Why bioproducts?

Industrial oil drilling began in the mid-nineteenth century, and in 2015, there was an assessed 35.2 billion barrels of proven oil reserves in the USA, with an estimated 3.4 billion barrels produced domestically that year [12]. While predictions of “peak oil” made over the past two decades have been overly pessimistic, it is not unreasonable to predict that recovery of global oil reserves will, at some point, become economically unfeasible [13]. Thus, an alternative renewable source of energy and substitutes for products derived from petroleum will be required in the future. Lignocellulosic biomass represents one of the few sources of renewable carbon, while renewable energy (e.g., electricity) can be generated from other renewable sources such as wind and solar power. Biorefineries can convert biomass into a range of products by employing integrated catalytic, thermochemical, and biological conversion processes that efficiently utilize the carbon and energy stored in that biomass [14, 15]. A future that does not rely on fossil resources will involve renewable electrical production paired with the generation of biomass-derived products.

The last two decades of research in carbohydrate conversion techniques have witnessed successful biofuel and biochemical production, but the conversion of the lignin fraction of biomass has been less explored [16]. Federal regulation and clean energy initiatives are targeting production rates of 79 billion liters per year of second-generation biofuels by 2022, and the quantity of lignin remaining after the sugar fraction is fermented to reach that target could be as high as 62 million dry tons annually [17–19]. Increasing lignin utilization would not only help offset the environmental impact of biomass refinement, but also drastically increase the economic feasibility of the biorefinery. Second-generation biofuels and value-added bioproducts derived from biomass represent versatile end products, but the future commercial viability of biorefinery products depends on efficient use of both the carbohydrate and lignin substrates [20].

## What is lignin?

Lignin is a complex and heterogeneous macromolecule composed of cross-linked aromatic monomers and imparts a rigid or “woody” characteristic of plants that helps provide structural support and limit degradation of polysaccharides. The molecular structure of lignin polymers is primarily derived from *p*-coumaryl, coniferyl, and sinapyl alcohols and corresponds to *p*-hydroxyphenyl, guaiacyl, and syringyl monolignol units, respectively; however, there are a variety of other units which occur less frequently [8, 21]. The composition and relative abundance of each type of monolignol varies species to species, genotype to genotype, across tissue types, between cell wall layers, across different development stages, and as a function of environmental factors [22]. The monolignols form several inter-monomer linkages, most commonly aryl ether bonds (e.g., β-*O*-4, α-*O*-4, and 4-*O*-5) [23]. The extremely diverse and variable molecular structure of lignin makes commercial degradation difficult and widespread utilization of lignin challenging; however, effective utilization of lignin is necessary for lignocellulose conversion profitability [24].

## Thermochemical and catalytic conversion of lignin

Research has been conducted to develop conversion technologies that deconstruct lignin, in particular lignin generated as a by-product of papermaking and biomass-derived carbohydrate fermentation, for the production of renewable fuels, chemicals, and materials [25]. Lignin’s inherent recalcitrance toward deconstruction makes it difficult to depolymerize for industrial purposes [19, 26]. Transforming lignin into higher-value products is further complicated by its structural diversity and the high propensity of its intermediates to engage in secondary reactions. An identical lignin conversion process can generate different distributions of compounds depending on the chemical and molecular structure of the lignin feedstock. Additionally, each type of conversion technology has numerous processing conditions that determine the product phase (i.e., solid, liquid, or gas), composition, and application, as well as other conversion performance metrics (e.g., product yield, productivity, selectivity, and composition). For example, a liquid fuel product can easily be derived from lignin using pyrolysis, which exposes the lignin to high temperatures in the absence of oxygen [27]. However, the chemical composition of this lignin-derived pyrolysis oil has such a wide distribution of compounds that it has little to no utility for chemical production [28].

Thermochemical and catalytic conversion technologies for lignin valorization primarily include pyrolysis, hydrothermal liquefaction (HTL), gasification, oxidative cracking, hydrogenolysis, and solvolysis (Table 1) [25, 29, 30]. These lignin conversion technologies generate gas, liquid, and/or solid breakdown products through numerous complex reactions. Due to differing process conditions defining these conversion technologies, certain reaction pathways are favored, which alters the yield and composition of the breakdown products. Catalytic technologies (i.e., oxidative cracking, hydrogenolysis, and solvolysis), which have been reviewed in detail by Zakzeski et al., provide a promising avenue to convert lignin selectively into its constituent monomers or monomer derivatives [30]. Aromatic carbon–oxygen bonds in aryl ether inter-monomer linkages, which comprise 50–60% of the inter-monomer linkages of lignin [31], represent a potential macromolecular “weak” point that could prove an effective target for selective depolymerization. In this case, a catalyst not only facilitates cleavage of specific bonds along the lignin chains, but also allows the cleavage to occur with a lower energy input, reducing the occurrence of secondary reactions with higher activation energies. The resulting product mixture would therefore have a much narrower distribution of aromatic compounds, which may be more amenable to cost-effective chemical separation and/or downstream upgrading.

**Table 1:** Summary of thermochemical and catalytic technologies for lignin conversion [25]

Technology	Main product	Product application	Process notes
Gasification	Syngas (gas)	Production of energy, hydrogen, and methanol (methanol synthesis); alkanes (Fischer–Tropsch); isobutane (isosynthesis); ethanol (fermentation and catalysts); aldehydes and alcohols (oxosynthesis)	Performed under high temperatures (> 700 °C); can involve the addition of water and catalyst
Fast pyrolysis/hydrothermal liquefaction (HTL)	Bio-oil (liquid)	Production of energy and various liquid fuels (e.g., biogasoline) by catalytic upgrading	Performed at 250 to 700 °C; can involve the addition of water (HTL), hydrogen (hydropyrolsis) and catalyst (catalytic pyrolysis)
Torrefaction/slow pyrolysis	Biochar (solid)	Used as a more optimized solid fuel for combustion	Performed at 200 to 350 °C
Solvolysis	Soluble lignin fragments (liquid)	Phenolics and alkyl phenolics	Two main categories (A) Acid- and base-catalyzed depolymerization (B) Supercritical solvent depolymerization
Hydrogenolysis	Soluble lignin fragments (liquid)	Phenolics and alkyl phenolics	Hydrogen donor (e.g., hydrogen gas, alcohol, or acid) and a catalyst can be used to cleave linkages
Oxidative cracking	Soluble lignin fragments (liquid)	Aromatic aldehyde, ketones, and carboxylic acids	Linkages in lignin can be cleaved by an oxidant (e.g., air and hydrogen peroxide) and a catalyst

## Biological and hybrid conversion of lignin

Although catalysts can provide a route toward selective lignin depolymerization chemistry, thermocatalytic processing of lignin often results in a product mixture that still requires extensive chemical separations. Additionally, lignin depolymerization products are generally limited to aromatic and phenolic derivatives. There has been significant research studying the application of enzymes and various microorganisms as a more selective and facile method of lignin depolymerization, as discussed in a recent report [32]. In general, biological systems require mild conditions that avoid costs associated with the use of high temperatures and high pressures. However, only a few bacteria (e.g., *Streptomyces* spp., *Rhodococcus* spp., and *Nocardia* spp.) and brown/white-rot fungi [33, 34] have an ability to depolymerize lignin, and their lignin depolymerization rate is too low to be useful on an industrial scale [26, 35].

To overcome these challenges, researchers have adopted a hybrid conversion approach which combines the best attributes of thermocatalytic and biological conversion technologies [36]. In a hybrid conversion approach, a thermocatalytic conversion process with advantageous reaction kinetics and conversion is applied for the initial lignin depolymerization. Downstream, microbial conversion and funneling of the depolymerized lignin breakdown products (LBPs) to a value-added product then occur with advantageous selectivity [37]. There are numerous aromatic catabolic pathways in various microbes which can be harnessed into a “biological funnel” by converting the heterogeneous substrates generated during depolymerization into common metabolic intermediates (e.g., protocatechuate and catechol) [38]. These intermediates undergo further conversion to central metabolites (e.g., acetyl-CoA) that can be utilized to produce target compounds at a high selectivity.

Hybrid conversion technologies have been previously implemented, but they have almost exclusively focused on sugar utilization [39, 40]. For example, cellulosic technologies can consist of thermochemical polysaccharide depolymerization (e.g., acid hydrolysis [41] or production of pyrolytic sugars [42]) and biological conversion of the resulting monosaccharides into ethanol or other products [39]. Recent work has begun to shift the focus from sugar fermentation to lignin utilization, with most research concentrating on using lignin model compounds to characterize aromatic degradation pathways [43–45] and bioconversion abilities [46, 47]. Demonstrations of an integrated thermochemical process with an aromatic-metabolizing microbial catalyst using pretreatment liquors have been performed, but these feedstocks frequently contain only a portion of the original lignin content, as the pretreatment process has been optimized for sugar release via enzymatic hydrolysis rather than maximizing lignin conversion [38, 48, 49]. For an effective lignin hybrid conversion process, the upstream thermochemical or catalytic depolymerization process must meet the following requirements: (1) production of aqueous soluble LBPs; (2) optimization of lignin conversion for yield and selectivity toward the preferred substrates for microbial growth and utilization; (3) minimal generation of inhibitor compounds; and (4) a process configuration and condition that is compatible with an economical, sustainable, and large-scale design. To this end, multiple lignocellulosic biomass pretreatment techniques have been tested, demonstrating the potential of hybrid conversion processes [48–52].

## Why *Rhodococcus opacus* PD630 for the hybrid conversion of lignin?

*Rhodococcus opacus* PD630 has been identified as a candidate biological catalyst for the conversion of both the carbohydrate and lignin fractions of lignocellulose into valuable products. *R. opacus* was originally isolated from soil collected near a gas works plant by enrichment on phenyldecane as a sole carbon source [11]. *R. opacus* possesses extensive catabolic pathways for both sugars and aromatics and can tolerate inhibitory compounds found in depolymerized biomass (e.g., phenolics and furfural) [53]. The ability to metabolize aromatic compounds is shared by a number of microorganisms and is likely a common evolutionary trait due to the prevalence of lignin in natural environments. Many of the aromatic compounds that *R. opacus* is known to metabolize can be found in LBPs [9, 54–58]. In addition to lignin model compounds (e.g., 4-hydroxybenzoate, benzoate, phenol, vanillate, guaiacol, and trans-*p*-coumaric acid), *R. opacus* has been shown to degrade depolymerized kraft lignin [59], alkali-treated corn stover [60, 61], alkali-treated poplar wood [34], and switchgrass pyrolysis oil [52]. Through adaptive evolution, *R. opacus* has been further evolved to more efficiently degrade phenol, syringaldehyde, and aromatic mixtures [9, 53, 54]. *R. opacus* has also been engineered using exogenous genes expressed on plasmids to degrade cellulose, arabinose, and xylose [62–64]. It is therefore, through native or engineered means, able to tolerate and utilize a variety of typically toxic lignin-derived compounds, in addition to sugars.

Unlike most bacteria that store carbon as polyhydroxyalkanoic acids (PHAs), *R. opacus* stores carbon as energy-rich triacylglycerols (TAGs) [58, 65]. Acetyl-CoA is the product of diverse catabolic pathways in *R. opacus*, including glycolysis, the Entner–Doudoroff pathway, and aromatic degradation pathways (e.g., β-ketoadipate pathway), and it is a key precursor in TAG biosynthesis. Under nitrogen limitation, the non-limiting essential nutrient (i.e., carbon) is stored as TAGs in *R. opacus*, accumulating up to ~ 80% of its cellular dry weight when cultured on gluconate [11, 66]. On aromatic compounds, lipid production is reduced, but accumulation can still reach up to 44% of cellular dry weight in TAGs under nitrogen-limiting conditions [9]. *R. opacus* can also synthesize branched-chain and odd-numbered fatty acids that are necessary for next-generation biofuels. Shifting lipid storage in *R. opacus* to these compounds would make it an even more valuable production strain [67].

Other organisms have been proposed for lignin conversion, but *R. opacus* has demonstrated equal or higher rates of aromatic degradation and tolerance compared to those species. For example, a phenol-adapted *Pseudomonas putida* strain had a maximum phenol degradation rate of ~ 12 mg phenol/L/h when grown at its maximum tolerated phenol concentration of 1 g/L [68]. *Bacillus brevis* previously claimed the highest phenol tolerance and utilization when cultures grew at concentrations up to 1.75 g/L phenol, and demonstrated a maximum degradation rate of ~ 20 mg/L/h [69]. Adapted *R. opacus* strains were able to grow at 2 g/L phenol and demonstrated a maximum degradation rate of ~ 21–22 mg/L/h [54].

In summary, *R. opacus* is an ideal candidate for the hybrid approach of lignocellulose utilization because of its high tolerance to aromatic compounds, its capacity to utilize a wide variety of substrates (both carbohydrates and LBPs), and its ability to accumulate lipids. These traits are uniquely suited to handling the diverse feedstock mixtures produced by lignocellulose depolymerization processes and metabolizing them into valuable compounds. Additionally, *R. opacus* is amenable to adaptive evolution to improve the tolerance and growth rate on aromatic and lignin substrates [9, 53, 54]. These natural characteristics, along with a growing toolbox of genetic tools, make *R. opacus* an ideal organism for lignin valorization [70].

## Genetic and metabolic characteristics of *R. opacus*

**Fig. 1:**
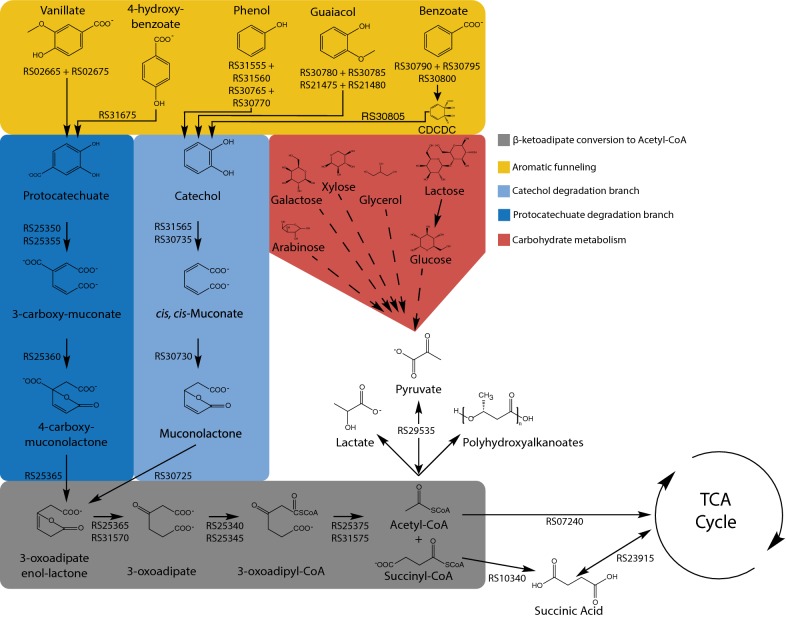
Aromatic degradation and carbon metabolism in *R. opacus*. *R. opacus* genes involved in reactions are listed. Dashed arrows represent multiple intermediate steps not shown. Xylose and arabinose consumptions occur via engineered pathways

Aromatic degradation in *R. opacus* is facilitated by a high-flux β-ketoadipate pathway that produces acetyl-CoA (Fig. 1) [71]. As acetyl-CoA is the precursor molecule for many biochemicals, *R. opacus* is thus well suited for chemical production based on an aromatic feedstock. Additionally, glucose metabolism in *R. opacus* exclusively utilizes the Entner–Doudoroff pathway, enabling simultaneous utilization of phenol and glucose. This lack of catabolite repression means that *R. opacus* can effectively use both the carbohydrate and lignin fractions of lignocellulosic biomass with reduced fermentation times and increased productivities. While sugar and phenol metabolisms are independent, *R. opacus* degrades aromatic compounds in a preferential order [9, 72]. It is unclear what is driving this preferential consumption, but it may result from variations in enzyme and transporter activities, or transcriptional-level regulation.

Aromatic compounds entering the cell generally first undergo preliminary degradation to either protocatechuate or catechol before being metabolized through the β-ketoadipate pathway. Mechanistically, this import into the cell and preprocessing occurs via specialized aromatic transporters and funneling enzymes, which have been identified using transcriptomics and proteomics [9, 34, 54]. Henson et al. identified three aromatic-associated transporters: one specific to phenol, one associated with both phenol and vanillate, and a promiscuous transporter associated with phenol, vanillate, benzoate, and guaiacol [9]. Several funneling enzymes have also been identified, including those which convert vanillate and 4-hydroxybenzoate to protocatechuate, as well as those which convert phenol, guaiacol, and benzoate to catechol [9].

Advantageous mutations and transcriptional changes have been identified in *R. opacus* that could be future targets for additional growth optimization through forward engineering. Genes for enzymes involved in oxidation–reduction reactions underwent changes in multiple strains adapted for improved growth on one or more phenolic compounds, including cytochrome ubiquinol oxidase subunit I and superoxide dismutase [9, 54]. The fact that functionally equivalent mutations occurred in multiple aromatic-adapted strains suggests their link to improved aromatic tolerance and utilization. For example, decreasing the activity of superoxide dismutase, as demonstrated in these mutated strains, may allow the cells to increase oxidizing equivalents, which are necessary to degrade highly reduced aromatic rings. Transcriptomic analysis of adapted strains also identified increased expression of aromatic transporters, which correlated with increased phenolic tolerance and utilization. These and other changes identified in the genome and transcriptome of adaptively evolved strains could be replicated in a rationally engineered strain to fine-tune its growth on and tolerance to aromatic-rich substrates.

Additional potential targets for strain optimization in *R. opacus* are its 9 endogenous plasmids (2 circular and 7 linear plasmids; a combined total of 0.79 Mbp). These plasmids have been posited to act as a hyper-recombinational gene storage strategy in which infrequently used catabolic genes are stored on plasmids as a failsafe against rarely encountered compounds (e.g., nitrophenolates and polycyclic and/or halogenated aromatics) which may be present in the environment [73–78]. If genes located on a plasmid are found to be regularly useful, they can undergo recombination with the 8.38 Mbp circular chromosome and become permanent components of the genome. This strategy has been observed in strains of related *Actinomycetales*, where stored genes provide the catabolic versatility to degrade a larger array of organic compounds [79, 80]. Additionally, genes located on plasmids, particularly if they are duplicates not subject to evolutionary conservation, can collect mutations more rapidly than those genes in the chromosome, allowing for improved adaptive capacity. For potential industrial use with a relatively well-defined and consistent feedstock, tailored strains of *R. opacus* may benefit from the selective removal of some of these plasmids, as previously adapted strains that exhibited improved growth profiles on phenolic compounds underwent large deletions or complete loss of plasmids 1 (0.17 Mbp) and 2 (0.098 Mbp) [9]. Plasmid removal under selective pressure may be driven by a reduced metabolic burden. Strategically, intentional plasmid curing would best be employed when cells are cultured on a defined range of carbon sources, where trading catabolic potential for improved growth rate is an acceptable risk.

## Tool and technique development for *R. opacus* engineering

As *R. opacus* is a non-model bacterium, the available genetic tools and techniques for engineering this organism were relatively sparse until recently. The genetic toolbox available for *R. opacus* has expanded to include a reference genome [58, 65], plasmid backbones for gene overexpression, and promoters for tunable gene expression (Table 2). Furthermore, methods for performing gene knockouts and knock-ins, modulating and quantifying gene expression, and extracting intracellular products via viral lysis have all been demonstrated. We summarize the most prominent genetic tools and techniques developed for several *R. opacus* strains.

**Table 2:** List of genetic parts demonstrated in *R. opacus*, including plasmid backbones, selection markers, promoters, and recombination-related parts

Part type	Name	Properties/notes	Source
Plasmid backbones	pAL5000 (short)	Other names: pXYLA and pNV18; ~ 11 copies per chromosome	[62, 73, 90–94]
	pAL5000 (long)	Other names: pJAM2 and pJEM; ~ 3 copies per chromosome	[63, 73, 90, 93]
	pNG2	Derived from *Corynebeacterium* spp.; ~ 10 copies per chromosome	[82, 84]
	pGA1	Derived from *Corynebeacterium* spp.	[63, 87, 95, 96]
	pSR1	Derived from *Corynebeacterium* spp.	[85, 86, 88]
	pB264	Derived from *Rhodococcus* sp. B264; curable; ~ 8 copies per chromosome	[73]
Selection markers	Kanamycin	50 μg/mL (selection) 250 μg/mL (plasmid function maintenance)	[85, 88, 97]
	Gentamicin	10 μg/mL	[82]
	Spectinomycin	100 μg/mL	[82]
	Thiostrepton	1 μg/mL	[97]
	Chloramphenicol	34 μg/mL	[98, 99]
	Hygromycin B	50 μg/mL	[73]
	SacB	Negative selection; sensitizes cell to sucrose	[100, 101]
Promoters	pTipA	Inducible with thiostrepton	[97, 102, 103]
	pAcet	5× inducible with acetamide	[98, 104]
	pBAD	59× inducible with arabinose	[104]
	pTet	67× inducible with anhydrotetracycline (aTc)	[104]
	pLPD06740	247× inducible with phenol	[104]
	pLPD06575	Inducible with phenol	[104]
	pLPD06699	Up to 39× inducible with phenol, protocatechuic acid, sodium benzoate, 4-hydroxybenzoate, vanillate, and guaiacol	[104]
	pLPD06568	Up to 80× inducible with phenol, sodium benzoate, and guaiacol	[104]
	pLPD03031	18× repressible with ammonium	[104]
	IGRI’ and IGRIV’	Inducible with 2,4-dinitrophenol (DNP)	[100]
Recombinases	Che9c60	GC-rich homologue of RecE	[73, 79, 105]
	Che9c61	GC-rich homologue of RecT	[73, 79, 105]
Neutral sites	ROCI-2	*R. opacus* chromosomal locus	[73]
	ROCI-3	*R. opacus* chromosomal locus	[73]
	ROP8I-1	*R. opacus* endogenous plasmid 8 locus	[73]

For promoters inducible with multiple chemicals, the highest published fold change for a single compound is reported

A common element used for gene overexpression is the replicating plasmid backbone. As previously mentioned, *R. opacus* PD630 has 9 endogenous plasmids, which range in size from 37 to 172 Kbp [19]. These endogenous plasmids have 1 to 5 copies per chromosome, with plasmid 8 (5 copies per chromosome) having a confirmed neutral site for stable integration of heterologous DNA constructs [73]. Additionally, there are a number of heterologous plasmids isolated from other *Actinomyces* spp. that have been demonstrated to replicate stably in *R. opacus* (Table 2). While they have several names, these plasmid backbones can be grouped into five primary categories. The pAL5000-based plasmid group, consisting of short and long variants, is derived from *Mycobacteria* spp. and has been demonstrated to have 3 to 11 copies per chromosome, dependent on the variant [73, 81, 82]. The pNG2, pGA1, and pSR1 plasmid groups are all ancestrally related and derived from cryptic *Corynebacterium* spp. plasmids that replicate through rolling-circle amplification [83–87]. A BioBrick-compatible version of pSR1 (pSRKBB) has recently been developed for easy cloning [88]. The pAL5000 and pNG2 backbones have been demonstrated to be compatible, allowing co-maintenance of two heterologous plasmids [73]. Finally, pB264, which is derived from an endogenous *Rhodococcus* sp. B264 plasmid, has ~ 8 copies per chromosome and is easily curable from the cell once antibiotic selection pressure is removed [73, 89]. To ensure that heterologous plasmids are stably maintained within a cell, selection is required, most frequently in the form of an antibiotic resistance marker. Several of these markers have been demonstrated and optimized for selection in *R. opacus* (Table 2), though there may be room for refinement with regard to stable maintenance concentrations [88]. A recent study employed single-cell fluorescence to demonstrate that the concentration of kanamycin sufficient for selection was not the ideal concentration for plasmid function maintenance. The commonly used concentration of 50 µg/mL led to a bimodal population of cell fluorescence, with fewer than half of the cells demonstrating fluorescent reporter expression when analyzed via flow cytometry [88]. Increasing the concentration of kanamycin to 250 µg/mL led to a majority of cells expressing the fluorescent reporter.

The ability to readily transform a bacterium with heterologous DNA is critical if it is to be a platform organism. *R. opacus* is transformable through multiple methods, including conjugation and electroporation. Conjugation requires a plasmid containing an origin of transfer (OriT) and a bacterial strain capable of conjugating with the strain of interest to horizontally transfer the plasmid [106]. Both *E. coli* DH5α-pKOS111-47 and *E. coli* S17.1 have been used as conjugative helper strains with *R. opacus* [98, 100]. Electroporation, wherein a pulse of electricity creates pores in the cellular membrane, can facilitate uptake of plasmid DNA in *R. opacus* at a reported efficiency of ~ 10^5^ CFUs/μg DNA [97, 107].

Successful cellular engineering requires the use of well-characterized genetic parts for predictable gene expression, and in non-model organisms, parts are often borrowed from related organisms [108]. One core component needed for reliable gene expression is the promoter, which drives gene transcription. A number of studies have utilized constitutive promoters from related gram-positive *Actinomycetales* (e.g., *Mycobacterium* spp. and *Streptomyces* spp.) or from genetically distant bacteria, such as gram-negative *E. coli*, for heterologous gene expression [62, 63, 82, 88, 96]. When performing metabolic engineering, however, a number of different promoters of varying strengths are required to balance the expression of multiple genes in an enzymatic pathway for optimal product titers [108, 109]. An alternative to using borrowed promoters is the creation of a de novo constitutive promoter library, where many promoters of varying strengths are developed and characterized. Using a fluorescent reporter, a constitutive promoter library spanning a 45-fold change in fluorescent output from weakest to strongest promoter was generated for *R. opacus* [73]. Performing initial optimization of an enzymatic pathway combinatorially with a range of constitutive promoters, however, can be time-consuming and costly in non-model organisms.

An alternative to constitutive promoters is tunable promoters whose expression is induced or repressed relative to the concentration of a specific compound. Table 2 summarizes the inducible and repressible promoters that have been demonstrated in *R. opacus*. Of particular interest to the goal of using *R. opacus* for the conversion of lignin to lipids are the aromatic- and ammonium-responsive promoters. The aromatic promoters (pLPD06740, pLPD06575, pLPD06699, pLPD06568) are differentially induced in the presence of a variety of aromatic compounds, including some found in depolymerized lignin, and could be employed in metabolic engineering related to aromatic catabolism [104]. pLPD03031 is a promoter that is repressed in the presence of ammonium, which can be used as a sole nitrogen source in *R. opacus*, and turns on when ammonium is depleted [104]. As nitrogen starvation triggers lipid accumulation in *R. opacus*, this promoter could be used to modulate lipid pathways under lipid-accumulating conditions [10, 54]. Combining the aromatic- and ammonium-responsive promoters into genetic circuits could lead to dynamic regulation, which has been shown to increase final product titers through reductions in metabolic burden [29]. Furthermore, pLPD03031 has been employed to create a cellular timer designed to activate at specific points in the cellular growth cycle, dependent on the initial ammonium concentration in the culture [104].

In addition to expressing a gene construct on a plasmid, heterologous expression can be achieved by integrating the DNA into the genome of the organism, where it can be stably maintained. To date, genome modification in *R. opacus* has been performed through both single- and double-crossover homologous recombination [73, 98, 100, 105]. Two methods utilizing single-crossover recombination, combined with the conjugative transfer of a donor plasmid via an *E. coli* helper strain, have been described for *R. opacus* [98, 100]. One difficulty with genomic recombination in *R. opacus*, however, is that it often results in illegitimate integration, wherein the integration cassette is inserted at an incorrect location or the entire plasmid is integrated. This is a common issue in other actinobacteria, such as *Mycobacterium tuberculosis* and *Rhodococcus fascians*, and can be overcome through the heterologous expression of helper recombinases [79, 110]. In *R. opacus*, a pair of bacteriophage recombinases, Che9c60 and Che9c61, has been demonstrated to facilitate double-crossover homologous recombination when donor template is provided via electroporation [73, 79, 105]. Ideally, the integration of foreign DNA into the genome would have no adverse effects on cell health, but in practice care must be taken in choosing an integration site. Three neutral sites, or locations that have been demonstrated not to cause a decrease in growth rate when a gene cassette is integrated into them, have been identified in the chromosome and a native endogenous plasmid of *R. opacus* [73].

In addition to the tools that facilitate gene overexpression in *R. opacus*, it may also be desirable to eliminate certain genes. Genetic knockouts through homologous recombination have also been performed to disrupt gene expression. Both single- and double-crossover recombination have been used to knockout and confirm the functional roles of transcriptional regulators, catabolic enzymes, and transporters in *R. opacus* [9, 100]. Furthermore, the *sacB* negative selection marker, which sensitizes the cell to sucrose, has been used for markerless genome engineering in *R. opacus* [100, 101].

Gene knockouts can be informative when investigating gene function, but as permanent modifications, they may be lethal or deleterious to the cell. As an alternative, a gene’s expression can be selectively and temporarily reduced through CRISPR interference (CRISPRi). CRISPRi utilizes a complex comprising a deactivated Cas9 nuclease (dCas9) and an engineered small guide RNA (sgRNA) to bind to DNA in a sequence-dependent manner and interfere with the transcriptional machinery, leading to targeted gene repression [111]. The most commonly used CRISPRi system is derived from *Streptococcus pyogenes* (dCas9_Spy_), but this system was found to be ineffective in *Mycobacterium tuberculosis*, an *Actinomycetales* species closely related to *R. opacus* [112]. A version of dCas9 sourced from *Streptococcus thermophilus* (dCas9_Sth1_), which was found to be effective in *M. tuberculosis*, has been developed as a repression system for use in *R. opacus* [73]. Experimentally, up to 58% repression of a chromosomally integrated fluorescent protein was observed using this optimized dCas9_Sth1_ [73]. Tunable gene repression using dCas9_Sth1_ can be used in the future to remodel native metabolic pathways in *R. opacus*.

To quantify changes in gene expression between different growth conditions, stably expressed reference (or “housekeeping”) genes in *R. opacus* have been identified for use with reverse transcription quantitative PCR (RT-qPCR) [113]. When ribosomal RNAs (rRNAs) are present in the samples, it was found that the combined use of genes for the ATP-binding subunit, ClpX, of the ATP-dependent Clp protease (PD630_RS25530) and 16S rRNA (PD630_RS01395) provided the best normalization results. If rRNAs are depleted, as is the case in samples prepared for RNA-Seq, the best pair of genes was found to be the same ATP-binding subunit ClpX and the rRNA small subunit methyltransferase G (PD630_RS37755). Using an appropriate set of reference genes is essential to generate meaningful expression-change data, and these pairs provide this baseline to an array of analyses.

A final technique that has been implemented to improve bioproduction in *R. opacus* is the development of a method for controlled cellular lysis to release intracellular compounds [114]. A bottleneck in microbial manufacturing is separating the target product from the cells, and implementing a controlled release strategy could reduce processing costs. A domesticated version of *R. opacus* created through serial culturing was found to be sensitive to a lytic tectivirus (Phage Toil) [114], which can be used to trigger cell lysis at a desired timepoint and can thus serve as a cheap and effective method for releasing products (e.g., TAGs) from *R. opacus* [114].

## *R. opacus* as a production host

The production of TAGs and fatty acids in *R. opacus* has been demonstrated on an array of carbon feedstocks (Table 3). When fed kraft lignin (a toxic by-product of the paper and pulping industry) in combination with laccase (a class of enzymes which oxidize phenolics), *R. opacus* was able to generate 0.145 g/L of lipid [59]. A strain of *R. opacus* adaptively evolved to tolerate higher levels of aromatic compounds, PVHG6, was able to generate 0.13 g/L lipids when provided with five lignin model compounds as sole carbon sources in equal quantities [9]. Growth of *R. opacus* on pre-treated corn stover produced 1.3 g/L of lipid (measured as fatty acid methyl esters (FAME)) [60]. A xylose-fermenting strain (MITXM-61) was developed by heterologous gene expression, and when it was grown in corn stover hydrolysates (containing 118 g/L initial total sugars), it converted xylose and glucose into 15.9 g/L TAGs [54% of dry cell weight (DCW)] [115]. When *R. opacus* was cultured in glucose and glycerol, Suwaleerat et al. observed a maximum of 2.4 g/L lipids in 10.2 g/L biomass [116]. While *R. opacus* grows poorly on glycerol alone [116, 117], this demonstrates that it can be used to enhance lipid production compared to just glucose as a sole carbon source. As glycerol is a by-product of TAG transesterification, feeding it back to the production strain could reduce overall costs [118]. Using adaptive evolution, Sinskey and colleagues generated an MITXM-61 derivative strain, MITGM-173, which was able to grow on up to 160 g/L glycerol [119]. Optimized TAG production in this adapted strain occurred with a 1:2:2 mixture of glycerol/glucose/xylose, reaching 13.6 g/L TAGs (51.2% of DCW).

**Table 3:** Bioproduction by *R. opacus* wild-type and engineered strains on various feedstocks

Strain	Substrate	Product	Production value	References
*R. opacus* PD630	glucose/glycerol (7:3)	Carotenoids and lipids	0.99 mg/L and 2.4 g/L, respectively	[116]
*R. opacus* PD630	Pre-treated corn stover	FAME	1.3 g/L	[60]
*R. opacus* PD630	Glycerol	TAGs	1.4 g/L, 38.4% DCW	[117]
*R. opacus* PD630 (engineered)	Glucose	Fatty acids	46% DCW	[120]
*R. opacus* MITXM-61 (engineered)	Corn stover hydrolysates	TAGs	15.9 g/L, 54% DCW	[115]
*R. opacus* MITGM-173 (evolved)	glycerol/ glucose/xylose (1:2:2)	TAGs	13.6 g/L, 51.2% DCW	[119]
*R. opacus* PD630	Crude whey	Fatty Acids	45.1% DCW	[121]
*R. opacus* PD630	Switchgrass pyrolysis oil	Lipid	pH 7: 0.078 g/L, 21.9% DCW pH 4: 0.066 g/L, 25.8% DCW	[52]
*R. opacus* PD630	Kraft lignin (+ laccase)	Lipid	0.145 g/L	[59]
*R. opacus* PD630	Olive mill waste	Lipid	~ 1.9 g/L, 80% DCW	[122]
*R. opacus* PD630 PVHG6	phenol/vanillate/4-hydroxybenzoate/guaiacol/benzoate (1:1:1:1:1)	Lipid	0.13 g/L, 44% DCW	[9]
*R. opacus* PD630 (engineered)	Gluconate and whey	Wax esters	Gluconate: 46% total neutral lipids Whey: NR	[99]
*R. opacus* PD630	Poplar lignin hydrolysis slurry	Lipid	NR	[34]

*FAME* fatty acid methyl ester, *DCW* dry cell weight, *NR* not reported

*Rhodococcus opacus* can also be used as a platform to produce high-value compounds other than lipids. For example, *R. opacus* naturally produces carotenoids, which are prized for their pigmentation and antioxidant properties [116]. In a 7:3 ratio of glucose and glycerol, *R. opacus* was able to produce 0.99 mg/L of carotenoids [116]. Though *R. opacus* has, at present, a limited pool of demonstrated products (i.e., lipids and carotenoids), there is potential to expand the range, particularly as its genetic toolbox has recently been developed. With rational metabolic engineering, it may be possible to shunt more carbon flux into carotenoid production to improve yields and titers. Similarly, manipulation of enzymes in the β-ketoadipate pathway (Fig. 1) could result in the accumulation of high-value intermediates, including *cis, cis*-muconic acid (bioplastic precursor) and succinic acid (food additive). Furthermore, the downstream product of aromatic degradation, acetyl-CoA, can be diverted to produce diverse compounds. Production of these high-value compounds from lignocellulose or lignocellulose-derived sources has been demonstrated in other bacterial hosts, which provides a guide for engineering *R. opacus* (Table 4).

**Table 4:** Compounds produced from lignin or lignin-derived sources in selected non-*R. opacus* bacterial hosts

Strain	Substrate	Product	Production value	Reference
*R. jostii* RHA1 (engineered)	Wheat straw	Vanillin	96 mg/L	[123]
*P. putida* KT2440 (engineered)	Depolymerized corn stover lignin	*Cis, cis*-muconic acid	3.7 g/L	[124]
*Corynebacterium glutamicum* (engineered)	Depolymerized softwood lignin	*Cis, cis*-muconic acid	1.8 g/L	[125]
*Cupriavidus basilensis* B-8	Kraft lignin	Polyhydroxyalkanoate (PHA)	319.4 mg/L	[126]
*Actinobacillus succinogenes* 130Z	Xylose-enriched corn stover hydrolysate	Succinic acid	39.6 g/L	[127]

## Conclusion

Biofuels and bioproducts can be produced from lignocellulose to replace or supplement petroleum-based fuels, chemicals, and materials. To improve the economic competitiveness and reduce the environmental footprint of biorefineries, both the carbohydrate and lignin fractions should be utilized. However, due to its recalcitrance, lignin has been an untapped carbon source which is either discarded or burned for process heat. Additionally, aromatic compounds found in depolymerized lignin are toxic to most microbes, presenting a challenge to developing an economically viable process. To overcome these challenges, we propose a hybrid conversion approach that combines thermochemical/catalytic and biological conversion processes as discussed in this review.

*Rhodococcus opacus* is an ideal organism for such a hybrid conversion process due to its ability to tolerate and utilize a wide variety of aromatic compounds found in lignin breakdown products. Additionally, *R. opacus* is oleaginous and can produce high levels of lipids. While *R. opacus* engineering has been limited, recent identification of aromatic degradation pathways and substrate transporters has provided several targets that can be modified for strain optimization. In addition, the toolbox for genetic engineering is under active development, providing methods for gene modification and transcription control. Further engineering will be necessary to increase the tolerance, growth rate, and lipid production of *R. opacus* on depolymerized lignin substrates. Furthermore, *R. opacus* can be engineered to synthesize more valuable chemicals, such as branched-chain fatty acid esters, carotenoids, and *cis, cis*-muconic acid. With advances in both lignin depolymerization processes and rational engineering tool development for *R. opacus*, the coming years will witness rapid progress toward cost-effective conversion of lignocellulose into bioproducts.

## Data Availability

Not applicable.

## Abbreviations

TAGs: triacylglycerols
HTL: hydrothermal liquefaction
LBPs: lignin breakdown products
PHA: polyhydroxyalkanoate
aTc: anhydrotetracycline
DNP: 2,4-dinitrophenol
OriT: origin of transfer
CRISPRi: CRISPR interference
dCas9: deactivated Cas9 nuclease
sgRNA: small guide RNA
dCas9_Spy_: dCas9 originating from *Streptococcus pyogenes*
dCas9_Sth1_: dCas9 originating from *Streptococcus thermophilus*
RT-qPCR: reverse transcription quantitative PCR
rRNA: ribosomal RNA
FAME: fatty acid methyl esters
DCW: dry cell weight
